# Visceral Adiposity Index in Type 2 Diabetes Mellitus (DM) and Its Correlation With Microvascular Complications

**DOI:** 10.7759/cureus.31279

**Published:** 2022-11-09

**Authors:** Vidyashree Hulkoti, Sourya Acharya, Samarth Shukla, Sunil Kumar, Ruchita Kabra, Apurva Dubey, Vivek Lahane, Anamika Giri

**Affiliations:** 1 Department of Medicine, Jawaharlal Nehru Medical College (JNMC) Datta Meghe Institute of Medical Sciences, Wardha, IND; 2 Department of Pathology, Jawaharlal Nehru Medical College (JNMC) Datta Meghe Institute of Medical Sciences, Wardha, IND; 3 Department of Internal Medicine, Jawaharlal Nehru Medical College (JNMC) Datta Meghe Institute of Medical Sciences, Wardha, IND

**Keywords:** visceral, obesity, complication, diabetes mellitus, anthropometric

## Abstract

Background

Obesity, specifically abdominal obesity, is a major risk factor for diabetes. A strong association has been marked between diabetes and obesity. Many abdominal obesity indices have been established, including waist circumference (WC), BMI, and a new tool, the visceral adiposity index (VAI). However, very limited research highlights the association of these anthropometric parameters and VAI to the various microvascular complications of diabetes mellitus (DM). The objective of this study is to investigate the association of VAI with microvascular complications such as retinopathy, nephropathy, and neuropathy in type 2 DM (T2DM) patients.

Methodology

Data from Acharya Vinoba Bhave Rural Hospital (AVBRH) was analyzed in this case-control study with a sample size of 250 patients consisting of 125 cases and 125 controls. The cases and controls were age- and gender-matched. BMI and WC were measured in these patients, and the VAI was calculated. These anthropometric parameters were then analyzed to estimate their correlation with the microvascular complications of T2DM.

Results

The mean age of cases in this study was 58.37 ± 12.08 years and that of controls was 57.61 ± 14.51 years. Anthropometric parameters, namely, BMI, WC, and VAI were raised in cases as compared with controls, and they showed significant statistical relation with diabetes (for BMI, *P *= 0.003; for WC, *P* = 0.001 for males and *P* = 0.002 for females; and for VAI, *P *= 0.005). A significant correlation was noted in the high-density lipoprotein (HDL) cholesterol (*P *= 0.017 for males and *P *= 0.0004 for females) and triglyceride (TG) levels (*P *< 0.0001) between cases and controls. On distributing the male and female cases in quartiles, it was observed that with increasing quartiles, VAI increased significantly and was associated with an increased risk of microvascular complications such as retinopathy, nephropathy, and neuropathy. When the anthropometric parameters and VAI were compared with the total microvascular complications and the receiver operating characteristic curve studied, VAI had the maximum AUC (AUC for VAI was 0.826, WC was 0.813, and BMI was 0.806). Univariate analysis of the various microvascular complications showed that WC, BMI, HDL, TGs, and glycated hemoglobin (HbA1c) were all significantly correlated to the microvascular complications in T2DM patients.

Conclusions

As the VAI was significantly raised in T2DM patients and also seen to be significantly associated with microvascular complications, it could be used as a screening tool for T2DM patients.

## Introduction

Diabetes is a major metabolic illness that affects people all over the world. According to the World Health Organization (WHO), around 537 million people (aged 20-79 years) worldwide had diabetes in 2021 [1]. By 2030, the overall number of individuals living with diabetes is expected to reach 643 million, and by 2045, it would reach 783 million. Nearly half of all cases related to diabetes are found in Southeast Asia and Western Pacific regions [1]. According to IDF diabetes statistics, type 2 diabetes mellitus (T2DM) affects 9.6% of individuals in India, with 53.1% of individuals having undiagnosed diabetes [1].

Diabetes has been continuously increasing over the last three decades, with huge increases in low- and middle-income countries. The obesity pandemic explains this projection, indicating that the estimated percentage of overweight or obese persons would reach 57.8% in 2030 worldwide [2-4].

Since 1975, obesity has almost tripled worldwide. It was noted that in 2016, more than 1.9 billion adults, aged 18 years or older, were overweight. Among these, over 650 million people were obese [5].

The BMI, waist-to-hip ratio, and waist circumference (WC) are all obesity indicators. Different components of body composition are represented by these indicators. The BMI measures the total body mass, while the WC and waist-to-height ratio show abdominal obesity. Furthermore, due to the protective benefits of muscle mass in those areas, a bigger hip and thigh circumference has been linked to a lower incidence of diabetes [6].

There is evidence that the sort of excess fat a person has is an important determinant of illness risk. Because visceral fat cells release proteins that contribute to inflammation, atherosclerosis, dyslipidemia, and hypertension, so visceral fat is more concerning than subcutaneous fat. As a result, visceral adipose tissue may be more closely linked to T2DM and its microvascular consequences than other obesity indicators [7,8].

Interestingly, it has been shown that visceral adiposity precedes the development of T2DM and exhibits an effect independent of fasting insulin, insulin secretion, glycemia, total and regional adiposities, and family history of diabetes [9].

Visceral adiposity index (VAI) is the product of WC, BMI, triglyceride (TG), and HDL levels [10] and is considered to be a measure of visceral fat.

Because visceral fat, as represented by VAI, starts an incremental trend even before the onset of diabetes; thus, it is of interest to study the VAI in diabetic patients and to compare them with healthy controls to understand the relationship between VAI and diabetes and its associated microvascular complications. Hence, this study was planned to evaluate the correlation of VAI in diabetes with its microvascular complications.

## Materials and methods

Study design and population

After getting ethical authorization, this cross-sectional study was conducted in Acharya Vinoba Bhave Rural Hospital (AVBRH), a tertiary care teaching hospital located in the rural area of Wardha District, over two years from August 2019 to September 2021. The study enlisted the participation of 250 people. This included 125 people with T2DM and 125 nondiabetic healthy controls who were age- and gender-matched. Ethical clearance was obtained from the Institutional Ethical Committee (IEC number, DMIMS(DU)/IEC/Aug-2019/8217).

Participants

Patients of T2DM, either newly diagnosed or previously diagnosed and on treatment with antidiabetic oral hypoglycemic agents or insulin and attending diabetes OPD or admitted to the Medicine Department, were taken as cases. Age- and gender-matched nondiabetic people admitted to the Medicine Department were taken as control subjects. Both these groups were interviewed, examined, investigated, and included in the study.

Study Group

Newly detected patients with diabetes were diagnosed as per the World Health Organization (WHO) criteria or patients already on either oral hypoglycemic agents (OHAs) or parenteral. Inclusion criteria are shown in Table 1.

**Table 1:** Inclusion criteria of the study.

Inclusion criteria
Age > 18 years
Both male and female subjects
Subjects capable of giving consent and voluntarily willing to participate in the study

Control Group

An equal number of age- and gender-matched nondiabetic healthy subjects were enrolled as controls. Exclusion criteria are shown in Table 2.

**Table 2:** Exclusion criteria of the study.

Exclusion criteria
Chronic kidney disease patients
Any nondiabetic nephropathy patients
Patients affected by hypertension
Patients affected by ocular disorders
Neuropathies of other etiologies like chronic liver disease patients, alcoholics, or drug addicts
Critically ill patients of type 2 diabetes mellitus
Patients who have not given consent

Anthropometric measurements

**Figure 1:**
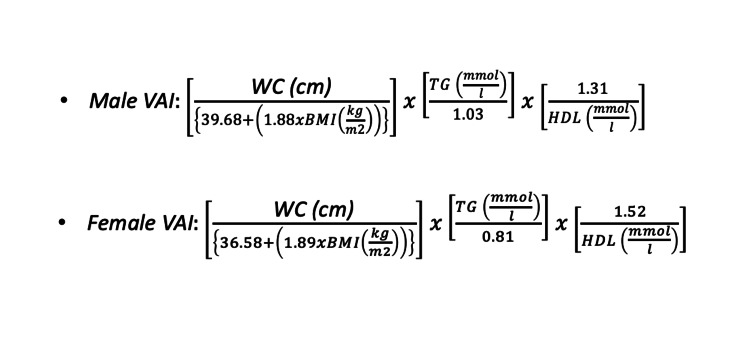
VAI formula. Source: [12]. VAI, visceral adiposity index; WC, waist circumference; TG, triglyceride; HDL, high-density lipoprotein; BMI, body mass index

BMI was computed using the formula weight/height squared (kg/m^2^), and weight (in kilograms) and height (in meters) were measured using established procedures. The participants were then classified using Asia Pacific criteria for BMI categorization [11]. For measuring the WC, the WHO STEPS protocol was utilized, which directs to measure at approximately halfway between the border of the last perceptible rib and the top of the iliac crest. For WC, the revised National Cholesterol Education Program, Adult Treatment Panel III (NCEP ATP III) Criteria for Asians were used. The VAI score was calculated, as described in Figure 1 [12], using the following sex-specific equations:

Biochemical measurements

Fasting and postprandial blood sugar levels were analyzed by the venous samples sent in fluoride bulbs and tested within 30 minutes after collection in RX Imola Analyzer from Randox Biosciences (Crumlin, UK) based on the colorimetric method without deproteinization. Hemoglobin A1c (HbA1c) was analyzed from the venous blood samples collected aseptically in dipotassium ethylenediaminetetraacetic acid (EDTA) bulbs and tested by the latex agglutination inhibition assay principle. For TGs and HDL, all the samples were processed by enzymatic method without correction of free glycerol using VITROS 5600 (Cardinal Health Ortho Clinical Diagnostics, Mumbai, India). Serum creatinine was evaluated by the serum of the sample using a centrifuge machine minimum of 10 minutes at 1,000-2,000 relative centrifugal force (RCF; generally 1,300 RCF) at room temperature. The estimated glomerular filtration rate (eGFR) was calculated from this serum creatinine using the Modification of Diet in Renal Disease Study Group (MDRD) Formula. Urine was examined to estimate microalbuminuria using the dipstick method. Fresh urine samples were collected in glass bulbs and tested within 1 hour at room temperature using Micral-Test (cobas; Roche Diagnostics, Indianapolis, IN, USA) urine test strips for microalbuminuria. A color change suggested a positive test, indicating microalbuminuria.

Examination parameters

Fundoscopy was done to evaluate for diabetic retinopathy. It was done by a traditional direct ophthalmoscope after dilating the pupil with mydriatic drops (tropicamide 0.5%), two drops 15-20 minutes before the examination and the drop application to be repeated every 30 minutes, as required. The retina is then visualized using the ophthalmoscope to check for changes in diabetic retinopathy. Nephropathy in diabetic patients is tested by microalbuminuria estimation in the urine sample, and eGFR is calculated. Monofilament was used to examine the sensory sensation, pain sensation was tested by a clean pin, a 128 Hz tuning fork was used to examine the vibration sense, and temperature sense was tested according to the neuropathy disability score (NDS) based on the Cochrane Systematic Review from the Cochrane Database [13], thereby examining the diabetic neuropathy. For motor examination, ankle jerk was examined.

Definitions

Diabetics: Patients with elevated blood sugar levels as per the WHO criteria for the diagnosis of diabetes or patients already on OHAs.

Criteria for the diagnosis of diabetes (WHO criteria [14]): Table 3 summarizes 2006 WHO recommendations for the diagnostic criteria for diabetes:

· Fasting plasma glucose ≥7.0 mmol/L (126 mg/dL), or

· Two-hour plasma glucose (means plasma glucose levels 2 hours after the oral glucose tolerance test [OGTT]) ≥ 11.1 mmol/L (200 mg/dL)

Overweight: Study participants will be classified based on their BMI as per WHO guidelines, as shown in Table 3 [11].

**Table 3:** Obesity showing WHO and Asia-Pacific guidelines. BMI, body mass index; WHO, World Health Organization

Parameters	WHO (BMI)	Asia-Pacific (BMI)
Underweight	<18.5	<18.5
Normal	18.5-24.9	18.5-22.9
Overweight	25-29.9	23-24.9
Obese	>=30	>=25

Micro-albuminuria: Microalbuminuria is defined as the excretion of more than 30 mg but less than 300 mg of albumin in the urine each day [15].

The albumin-to-creatinine ratio (ACR) is the preferred preliminary approach for detecting increased protein. The measurement of urinary ACR in a spot urine sample is the standard approach for evaluating albuminuria.

The albumin concentration (in milligrams) is divided by the creatinine concentration (in grams) to determine the ACR, as shown in Table 4 [15].

**Table 4:** Albuminuria categories in CKD. *Relative to young adult level. ACR = 30-300 mg/g for >3 months indicates CKD. **Including nephrotic syndrome (albumin excretion ACR > 2,220 mg/g). ACR, albumin-to-creatinine ratio; CKD, chronic kidney disease

Category	ACR (mg/g)	Terms
A1	<30	Normal to mildly increased
A2	30-300	Moderately increased*
A3	>300	Severely increased**

Data Analysis

The categorical variables were presented in the form of numbers and percentages. Quantitative data, on the other hand, was presented as mean ± SD and median with 25th and 75th percentiles (interquartile range). The Kolmogorov-Smirnov test was used to ensure that the data was normal. We used nonparametric tests in circumstances when data was not normal. The results were subjected to the following statistical tests:

· The quantitative variables that are not normally distributed in nature were analyzed using the Mann-Whitney test, and the independent t-test was used for comparison of normally distributed quantitative data. For the trend of nonnormally distributed parameters with the VAI quartile, the Jonckheere-Terpstra test was used, and for the trend of normally distributed parameters with the VAI quartile, one-way ANOVA with contrast was used.

· The chi-square test was used to compare the qualitative variables. Fisher's exact test was applied if any cell had an expected value of less than 5. The trend of qualitative parameters with the VAI quartile was determined using the chi-square test for trend.

· The cutoff values of the VAI, BMI (kg/m²), and waist-to-hip ratio for predicting nephropathy, neuropathy, retinopathy, and microvascular complications were calculated by the receiver operating characteristic curve.

· Univariate logistic regression was used to find out significant risk factors of retinopathy, nephropathy, neuropathy, and microvascular complications.

Data was entered into a Microsoft EXCEL spreadsheet, and the final analysis was performed using IBM's SPSS Statistics (version 21.0, Chicago, IL, USA).

A *P*-value of less than 0.05 was considered statistically significant.

## Results

The baseline characteristics of cases and controls are shown in Table 5. 

**Table 5:** The baseline characteristics of cases and control group. *Independent t-test. ^†^Mann-Whitney test. ^‡^Fisher's exact test. ^§^Chi-square test. SD, standard deviation; HDL, high-density lipoprotein; BMI, body mass index; HbA1c, glycated hemoglobin; VAI, visceral adiposity index

Parameters	Cases	Controls	Total	*P*-value
Age (years)				
18-30	1 (0.80%)	6 (4.80%)	7 (2.80%)	0.155^‡^
31-40	8 (6.40%)	10 (8%)	18 (7.20%)	
41-50	29 (23.20%)	21 (16.80%)	50 (20%)	
51-60	31 (24.80%)	35 (28%)	66 (26.40%)	
61-70	34 (27.20%)	28 (22.40%)	62 (24.80%)	
71-80	21 (16.80%)	19 (15.20%)	40 (16%)	
>80	1 (0.80%)	6 (4.80%)	7 (2.80%)	
Mean ± SD	58.37 ± 12.08	57.61 ± 14.51	57.99 ± 13.33	0.653*
Median (25th-75th percentile)	58 (49-68)	58 (50-69)	58 (50-68.75)	
Range	30-87	24-92	24-92	
Gender				
Female	55 (44%)	55 (44%)	110 (44%)	1§
Male	70 (56%)	70 (56%)	140 (56%)	
BMI (kg/m²)				
18.5-22.99 (normal)	61 (48.80%)	87 (69.60%)	148 (59.20%)	0.003§
23-24.99 (overweight)	46 (36.80%)	29 (23.20%)	75 (30%)	
>25 (obese)	18 (14.40%)	9 (7.20%)	27 (10.80%)	
Mean ± SD	22.96 ± 1.73	22.51 ± 1.67	22.73 ± 1.71	0.036*
Median (25th-75th percentile)	23.1 (21.7-24)	22.39 (21.457-23.437)	22.6 (21.5-23.7)	
Range	19.7-26.7	19.61-28.57	19.61-28.57	
Waist circumference (cm; male)				
<=90	59 (84.29%)	66 (94.29%)	125 (89.29%)	No P-value
>90	11 (15.71%)	4 (5.71%)	15 (10.71%)	
Mean ± SD	83.5 ± 6.77	80.04 ± 5.43	81.77 ± 6.36	0.001*
Median (25th-75th percentile)	84 (77-89.5)	79 (77-82)	80 (77-88)	
Range	72-96	72-92	72-96	
Waist circumference (cm; female)				
<=80 cm	30 (54.55%)	40 (72.73%)	70 (63.64%)	0.06‡
>80 cm	25 (45.45%)	15 (27.27%)	40 (36.36%)	
Mean ± SD	80.67 ± 5.99	77.13 ± 5.85	78.9 ± 6.16	0.002*
Median (25th-75th percentile)	80 (76-85.5)	77 (73.5-81.5)	78 (75-84)	
Range	69-91	65-92	65-92	
HDL (mmol/L; male)				
>=1.03	58 (82.86%)	66 (94.29%)	124 (88.57%)	0.06‡
<1.03	12 (17.14%)	4 (5.71%)	16 (11.43%)	
Mean ± SD	1.35 ± 0.32	1.48 ± 0.32	1.41 ± 0.32	0.017*
Median (25th-75th percentile)	1.3 (1.1-1.6)	1.38 (1.24-1.68)	1.33 (1.2-1.652)	
Range	0.8-2	0.9-2.12	0.8-2.12	
HDL (mmol/L; female)				
>=1.29	47 (85.45%)	55 (100%)	102 (92.73%)	0.006‡
<1.29	8 (14.55%)	0 (0%)	8 (7.27%)	
Mean ± SD	1.46 ± 0.24	1.62 ± 0.22	1.54 ± 0.25	0.0004*
Median (25th-75th percentile)	1.48 (1.3-1.6)	1.6 (1.49-1.7)	1.5 (1.4-1.7)	
Range	1-2	1.3-2.2	1-2.2	
Triglyceride (mmol/L)				
<1.69	89 (71.20%)	111 (88.80%)	200 (80%)	<0.0001
1.69 to 2.24	12 (9.60%)	12 (9.60%)	24 (9.60%)	
2.25 to 5.63	24 (19.20%)	2 (1.60%)	26 (10.40%)	
Mean ± SD	1.46 ± 0.76	1.19 ± 0.4	1.32 ± 0.62	0.088†
Median (25th-75th percentile)	1.2 (0.9-1.8)	1.2 (0.9-1.5)	1.2 (0.9-1.6)	
Range	0.6-3.6	0.6-2.3	0.6-3.6	
Retinopathy	72 (57.60%)	0	72 (28.80%)	<0.0001
Neuropathy	63 (50.40%)	0	63 (25.20%)	<0.0001
Nephropathy	75 (60%)	0	75 (30%)	<0.0001
Height(cm)				
Mean ± SD	166.62 ± 6.61	164.41 ± 8.58	165.51 ± 7.72	0.024†
Median (25th-75th percentile)	169 (160-172)	165 (158-170)	168 (159-172)	
Range	154-177	145-192	145-192	
Weight (kg)				
Mean ± SD	63.78 ± 6.4	60.96 ± 7.54	62.37 ± 7.12	0.002*
Median (25th-75th percentile)	63 (59-68)	61 (56-65)	62 (58-67)	
Range	50-80	44-90	44-90	
HbA1c (%)				
Mean ± SD	10.52 ± 2.62	5.53 ± 0.49	8.03 ± 3.13	<0.0001
Median (25th-75th percentile)	9.7 (8.4-13)	5.46 (5.13-5.9)	6.65 (5.462-9.675)	
Range	6.8-15.2	4.34-6.5	4.34-15.2	
VAI				
Mean ± SD	2.35 ± 1.74	1.56 ± 0.8	1.96 ± 1.41	0.005†
Median (25th-75th percentile)	1.74 (0.994-3.151)	1.37 (0.965-1.855)	1.51 (0.978-2.399)	
Range	0.52-6.89	0.54-4.01	0.52-6.89	

The association of various parameters such as age group, BMI, height, weight, WC, HDL, and TGs; microvascular complications such as retinopathy, nephropathy, and neuropathy; and blood parameters such as HbA1c with VAI among females in the study is shown in Table 6.

**Table 6:** Association of various parameters with the VAI quartile in cases (female). ^††^Chi-square test for trend. ¶One-way ANOVA with contrast. **Jonckheere-Terpstra test. SD, standard deviation; HDL, high-density lipoprotein; BMI, body mass index; HbA1c, glycated hemoglobin; VAI, visceral adiposity index

Parameters	First quartile (<0.9625; *n* = 14)	Second quartile (0.9625 to 1.496; *n* = 13)	Third quartile (1.497 to 2.609; *n* = 14)	Fourth quartile (>2.609; *n* = 14)	Total	*P*-value
Age (years)						
18-30	0 (0%)	0 (0%)	0 (0%)	1 (7.14%)	1 (1.82%)	0.688^††^
31-40	2 (14.29%)	0 (0%)	0 (0%)	1 (7.14%)	3 (5.45%)	
41-50	4 (28.57%)	5 (38.46%)	5 (35.71%)	1 (7.14%)	15 (27.27%)	
51-60	2 (14.29%)	4 (30.77%)	6 (42.86%)	5 (35.71%)	17 (30.91%)	
61-70	4 (28.57%)	3 (23.08%)	2 (14.29%)	3 (21.43%)	12 (21.82%)	
71-80	2 (14.29%)	1 (7.69%)	1 (7.14%)	3 (21.43%)	7 (12.73%)	
Mean ± SD	55.79 ± 12.3	54.85 ± 11.26	55.21 ± 8.03	58.5 ± 14.65	56.11 ± 11.57	0.550^¶^
Median (25th-75th percentile)	56 (45.75-63)	55 (45-63)	52 (50-59.25)	58 (53.5-69.5)	55 (47.5-65)	
Range	40-80	41-76	45-72	30-80	30-80	
BMI (kg/m²)						
18.5-22.99 (Normal)	9 (64.29%)	10 (76.92%)	3 (21.43%)	4 (28.57%)	26 (47.27%)	0.001^††^
23-24.99 (Overweight)	5 (35.71%)	3 (23.08%)	7 (50%)	4 (28.57%)	19 (34.55%)	
>25 (Obese)	0 (0%)	0 (0%)	4 (28.57%)	6 (42.86%)	10 (18.18%)	
Mean ± SD	21.96 ± 1.61	21.99 ± 1.01	24.14 ± 1.19	24.44 ± 1.63	23.15 ± 1.79	<0.0001
Median (25th-75th percentile)	22.45 (20.425-23.425)	21.9 (21.6-22.3)	24.2 (23.475-25.05)	23.95 (22.925-25.875)	23.2 (21.95-24.1)	
Range	19.7-23.9	20.4-23.7	22-25.8	22.6-26.7	19.7-26.7	
Waist circumference (cm)						
<=80	14 (100%)	11 (84.62%)	4 (28.57%)	1 (7.14%)	30 (54.55%)	0.003^††^
>80	0 (0%)	2 (15.38%)	10 (71.43%)	13 (92.86%)	25 (45.45%)	
Mean ± SD	75.07 ± 3.77	76.92 ± 3.12	84.36 ± 3.89	86.07 ± 4.12	80.67 ± 5.99	<0.0001
Median (25th-75th percentile)	75 (72.25-78)	76 (75-78)	85 (82.5-86.75)	87 (81.75-90)	80 (76-85.5)	
Range	69-80	73-83	77-90	80-91	69-91	
HDL (mmol/L)						
>=1.29	14 (100%)	13 (100%)	13 (92.86%)	7 (50%)	47 (85.45%)	0.0002^††^
<1.29	0 (0%)	0 (0%)	1 (7.14%)	7 (50%)	8 (14.55%)	
Mean ± SD	1.7 ± 0.2	1.59 ± 0.08	1.37 ± 0.08	1.19 ± 0.14	1.46 ± 0.24	<0.0001
Median (25th-75th percentile)	1.66 (1.515-1.852)	1.6 (1.5-1.6)	1.4 (1.3-1.4)	1.25 (1.025-1.3)	1.48 (1.3-1.6)	
Range	1.47-2	1.5-1.7	1.2-1.5	1-1.3	1-2	
Triglyceride (mmol/L)						
<1.69	14 (100%)	13 (100%)	14 (100%)	1 (7.14%)	42 (76.36%)	<0.0001
1.69 to 2.24	0 (0%)	0 (0%)	0 (0%)	4 (28.57%)	4 (7.27%)	
2.25 to 5.63	0 (0%)	0 (0%)	0 (0%)	9 (64.29%)	9 (16.36%)	
Mean ± SD	0.7 ± 0.05	1.08 ± 0.11	1.38 ± 0.18	2.4 ± 0.4	1.4 ± 0.68	<0.0001
Median (25th-75th percentile)	0.69 (0.68-0.732)	1.1 (1-1.2)	1.35 (1.225-1.575)	2.45 (2.125-2.7)	1.2 (0.845-1.6)	
Range	0.6-0.79	0.9-1.2	1.1-1.6	1.6-3	0.6-3	
Retinopathy	5 (35.71%)	6 (46.15%)	8 (57.14%)	13 (92.86%)	32 (58.18%)	0.002^††^
Neuropathy	5 (35.71%)	6 (46.15%)	7 (50%)	11 (78.57%)	29 (52.73%)	0.028^††^
Nephropathy	5 (35.71%)	6 (46.15%)	8 (57.14%)	14 (100%)	33 (60%)	0.001^††^
Height (cm)						
Mean ± SD	165.57 ± 4.03	159.77 ± 4.78	157.86 ± 1.79	158.57 ± 3.18	160.45 ± 4.67	<0.0001
Median (25th-75th percentile)	165.5 (162.25-169)	159 (156-161)	158.5 (157-159)	159.5 (156-160.75)	160 (157-162.5)	
Range	159-172	155-170	155-160	154-164	154-172	
Weight (kg)						
Mean ± SD	60.29 ± 6.21	56.15 ± 3.46	60.21 ± 3.42	61.43 ± 4.24	59.58 ± 4.8	0.170^¶^
Median (25th-75th percentile)	62 (56-65)	56 (54-59)	60 (57.5-62.75)	60.5 (58.25-63)	59 (56-63)	
Range	50-68	50-63	55-66	55-70	50-70	
HbA1c (%)						
Mean ± SD	7.8 ± 0.73	9.19 ± 0.88	11.17 ± 1.29	14.32 ± 0.52	10.65 ± 2.64	<0.0001
Median (25th-75th percentile)	7.7 (7.225-8.35)	9.1 (8.5-9.7)	11.35 (10.35-11.875)	14.3 (13.925-14.775)	10 (8.45-13.25)	
Range	6.8-9.1	8.1-11	9-13	13.5-15	6.8-15	
VAI						
Mean ± SD	0.75 ± 0.12	1.27 ± 0.18	1.96 ± 0.38	4.06 ± 1.12	2.02 ± 1.41	<0.0001
Median (25th-75th percentile)	0.77 (0.666-0.844)	1.38 (1.131-1.404)	1.85 (1.645-2.351)	3.86 (3.121-5.155)	1.5 (0.963-2.609)	
Range	0.52-0.96	0.97-1.49	1.5-2.59	2.63-5.82	0.52-5.82	

The association of various parameters such as age group, BMI, height, weight, WC, HDL, and TGs; microvascular complications such as retinopathy, nephropathy, and neuropathy; and blood parameters such as HbA1c with VAI among males in the study is shown in Table 7.

**Table 7:** Association of various parameters with the VAI quartile in cases (male). ^††^Chi-square test for trend. ^¶^One-way ANOVA with contrast. ^**^Jonckheere-Terpstra test. SD, standard deviation; HDL, high-density lipoprotein; BMI, body mass index; HbA1c, glycated hemoglobin; VAI, visceral adiposity index; WC, waist circumference

Parameters	First quartile (<1.061; *n* = 18)	Second quartile (1.061-1.9476; *n* = 17)	Third quartile (1.9477-3.5101; *n* = 17)	Fourth quartile (>3.5101; *n* = 18)	Total	P-value
Age (years)						
31-40	0 (0%)	5 (29.41%)	0 (0%)	0 (0%)	5 (7.14%)	0.085^††^
41-50	3 (16.67%)	4 (23.53%)	5 (29.41%)	2 (11.11%)	14 (20%)	
51-60	6 (33.33%)	3 (17.65%)	2 (11.76%)	3 (16.67%)	14 (20%)	
61-70	5 (27.78%)	4 (23.53%)	6 (35.29%)	7 (38.89%)	22 (31.43%)	
71-80	4 (22.22%)	1 (5.88%)	4 (23.53%)	5 (27.78%)	14 (20%)	
>80	0 (0%)	0 (0%)	0 (0%)	1 (5.56%)	1 (1.43%)	
Mean ± SD	61.5 ± 10.38	51.65 ± 12.81	60.35 ± 11.72	66.61 ± 9.95	60.14 ± 12.25	0.047^¶^
Median (25th-75th percentile)	60.5 (53.5-68.75)	49 (40-61)	63 (50-70)	69.5 (59.5-73.75)	61 (50-70)	
Range	45-78	33-72	44-80	49-87	33-87	
BMI (kg/m²)						
18.5-22.99 (Normal)	18 (100%)	10 (58.82%)	7 (41.18%)	0 (0%)	35 (50%)	<0.0001
23-24.99 (Overweight)	0 (0%)	7 (41.18%)	9 (52.94%)	11 (61.11%)	27 (38.57%)	
>25 (Obese)	0 (0%)	0 (0%)	1 (5.88%)	7 (38.89%)	8 (11.43%)	
Mean ± SD	20.84 ± 0.84	22.44 ± 1.1	23.39 ± 1.01	24.56 ± 0.9	22.8 ± 1.68	<0.0001
Median (25th-75th percentile)	20.7 (20.225-21.5)	22.6 (21.6-23.5)	23.5 (22.5-24)	24.4 (23.825-25.275)	22.95 (21.5-23.875)	
Range	19.7-22.3	20.4-23.8	22.1-25.5	23.4-26.3	19.7-26.3	
WC (cm)						
<=90	18 (100%)	17 (100%)	16 (94.12%)	8 (44.44%)	59 (84.29%)	No P-value
>90	0	0	1 (5.88%)	10 (55.56%)	11 (15.71)	
Mean ± SD	75.44 ± 3.68	80.76 ± 3.36	87 ± 2.72	90.83 ± 3.11	83.5 ± 6.77	<0.0001
Median (25th-75th percentile)	74.5 (73-76)	81 (77-84)	88 (85-88)	91 (90-93)	84 (77-89.5)	
Range	72-83	76-85	82-92	84-96	72-96	
HDL (mmol/L)						
>=1.03	18 (100%)	17 (100%)	16 (94.12%)	7 (38.89%)	58 (82.86%)	<0.0001
<1.03	0 (0%)	0 (0%)	1 (5.88%)	11 (61.11%)	12 (17.14%)	
Mean ± SD	1.78 ± 0.15	1.41 ± 0.11	1.18 ± 0.08	1.01 ± 0.14	1.35 ± 0.32	<0.0001
Median (25th-75th percentile)	1.75 (1.7-1.9)	1.4 (1.3-1.5)	1.2 (1.1-1.2)	1 (0.9-1.1)	1.3 (1.1-1.6)	
Range	1.6-2	1.3-1.6	1-1.3	0.8-1.21	0.8-2	
Triglyceride (mmol/L)						
<1.69	18 (100%)	17 (100%)	12 (70.59%)	0 (0%)	47 (67.14%)	<0.0001
1.69-2.24	0 (0%)	0 (0%)	5 (29.41%)	3 (16.67%)	8 (11.43%)	
2.25-5.63	0 (0%)	0 (0%)	0 (0%)	15 (83.33%)	15 (21.43%)	
Mean ± SD	0.69 ± 0.09	1.08 ± 0.13	1.52 ± 0.18	2.71 ± 0.5	1.5 ± 0.82	<0.0001
Median (25th-75th percentile)	0.68 (0.61-0.7)	1.1 (1-1.2)	1.5 (1.4-1.7)	2.75 (2.325-3)	1.2 (0.9-1.8)	
Range	0.6-0.9	0.9-1.3	1.2-1.8	1.8-3.6	0.6-3.6	
Retinopathy	6 (33.33%)	8 (47.06%)	10 (58.82%)	16 (88.89%)	40 (57.14%)	0.001^††^
Neuropathy	5 (27.78%)	7 (41.18%)	9 (52.94%)	13 (72.22%)	34 (48.57%)	0.006^††^
Nephropathy	6 (33.33%)	8 (47.06%)	10 (58.82%)	18 (100%)	42 (60%)	<0.0001
Height (cm)						
Mean ± SD	171.78 ± 3.3	171 ± 2.24	171 ± 2.55	172 ± 2.72	171.46 ± 2.72	0.886^**^
Median (25th-75th percentile)	172.5 (169.25-174)	170 (169-173)	170 (169-173)	172 (170-174.5)	171.5 (169-173)	
Range	165-176	168-176	168-177	168-176	165-177	
Weight (kg)						
Mean ± SD	61.5 ± 3.09	65.65 ± 3.95	68.47 ± 4.03	72.67 ± 3.77	67.07 ± 5.52	<0.0001
Median (25th-75th percentile)	62 (61-63.75)	67 (62-68)	68 (66-71)	73 (69.25-75.75)	67 (63-70)	
Range	54-65	58-71	63-80	66-79	54-80	
HbA1c (%)						
Mean ± SD	7.76 ± 0.8	9.08 ± 0.77	11.06 ± 1.46	13.77 ± 1.79	10.43 ± 2.62	<0.0001
Median (25th-75th percentile)	7.6 (7.125-8.35)	9 (8.5-9.6)	11 (10.2-11.9)	14.05 (13.825-14.775)	9.5 (8.4-12.95)	
Range	6.8-9.3	8.1-11	8.3-13.1	8.9-15.2	6.8-15.2	
VAI						
Mean ± SD	0.73 ± 0.17	1.5 ± 0.29	2.63 ± 0.45	5.53 ± 0.94	2.61 ± 1.93	<0.0001
Median (25th-75th percentile)	0.69 (0.61-0.769)	1.51 (1.251-1.784)	2.51 (2.354-2.966)	5.6 (5.056-6.166)	1.95 (1.061-3.51)	
Range	0.55-1.06	1.07-1.93	1.96-3.31	3.58-6.89	0.55-6.89	

Microvascular complications

The association of various parameters such as age, gender, height, weight, WC, BMI, HDL, TGs, HbA1c, and VAI with microvascular complications is shown in Table 8.

**Table 8:** Association of various parameters with microvascular complications. ^*^Independent *t*-test. ^†^Mann-Whitney test. ^‡^Fisher's exact test. ^§^Chi-square test. SD, standard deviation; HDL, high-density lipoprotein; BMI, body mass index; HbA1c, glycated hemoglobin; VAI, visceral adiposity index; WC, waist circumference

Parameters	Without microvascular complications	With Microvascular complications	Total	P-value
Age (years)				
18-30	0 (0%)	1 (1.20%)	1 (0.80%)	0.298^‡^
31-40	5 (11.90%)	3 (3.61%)	8 (6.40%)	
41-50	12 (28.57%)	17 (20.48%)	29 (23.20%)	
51-60	9 (21.43%)	22 (26.51%)	31 (24.80%)	
61-70	12 (28.57%)	22 (26.51%)	34 (27.20%)	
71-80	4 (9.52%)	17 (20.48%)	21 (16.80%)	
>80	0 (0%)	1 (1.20%)	1 (0.80%)	
Mean ± SD	55.17 ± 11.28	59.99 ± 12.21	58.37 ± 12.08	0.034^*^
Median (25th-75th percentile)	55 (47-63.75)	60 (51-70)	58 (49-68)	
Range	33-77	30-87	30-87	
Gender				
Female	15 (35.71%)	40 (48.19%)	55 (44%)	0.184^§^
Male	27 (64.29%)	43 (51.81%)	70 (56%)	
BMI (kg/m²)				
18.5-22.99 (Normal)	33 (78.57%)	28 (33.73%)	61 (48.80%)	<0.0001
23-24.99 (Overweight)	9 (21.43%)	37 (44.58%)	46 (36.80%)	
>25 (Obese)	0 (0%)	18 (21.69%)	18 (14.40%)	
Mean ± SD	21.7 ± 1.52	23.6 ± 1.47	22.96 ± 1.73	<0.0001
Median (25th-75th percentile)	21.55 (20.4-22.675)	23.5 (22.55-24.45)	23.1 (21.7-24)	
Range	19.7-24.6	20.4-26.7	19.7-26.7	
WC (cm; male)				
<=90	27 (100%)	32 (74.42%)	59 (84.29%)	
>90	0	11 (25.58%)	11 (15.71%)	
Mean ± SD	78.07 ± 5.15	86.91 ± 5.3	83.5 ± 6.77	<0.0001
Median (25th-75th percentile)	76 (73.5-82.5)	88 (84-90.5)	84 (77-89.5)	
Range	72-88	75-96	72-96	
WC (cm; female)				
<=80	11 (73.33%)	19 (47.50%)	30 (54.55%)	0.171^‡^
>80	4 (26.67%)	21 (52.50%)	25 (45.45%)	
Mean ± SD	76.6 ± 5.45	82.2 ± 5.51	80.67 ± 5.99	0.001^*^
Median (25th-75th percentile)	75 (72.5-81)	81 (77.75-87)	80 (76-85.5)	
Range	69-85	73-91	69-91	
HDL (mmol/L; male)				
>=1.03	27 (100%)	31 (72.09%)	58 (82.86%)	0.002^‡^
<1.03	0 (0%)	12 (27.91%)	12 (17.14%)	
Mean ± SD	1.58 ± 0.28	1.2 ± 0.25	1.35 ± 0.32	<0.0001
Median (25th-75th percentile)	1.5 (1.35-1.8)	1.2 (1-1.3)	1.3 (1.1-1.6)	
Range	1.2-2	0.8-1.7	0.8-2	
HDL (mmol/L; female)				
>=1.29	15 (100%)	32 (80%)	47 (85.45%)	0.091^‡^
<1.29	0 (0%)	8 (20%)	8 (14.55%)	
Mean ± SD	1.67 ± 0.22	1.38 ± 0.2	1.46 ± 0.24	<0.0001
Median (25th-75th percentile)	1.7 (1.48-1.78)	1.4 (1.3-1.5)	1.48 (1.3-1.6)	
Range	1.4-2	1-1.83	1-2	
Triglyceride (mmol/L)				
<1.69	42 (100%)	47 (56.63%)	89 (71.20%)	<0.0001
1.69-2.24	0 (0%)	12 (14.46%)	12 (9.60%)	
2.25-5.63	0 (0%)	24 (28.92%)	24 (19.20%)	
Mean ± SD	0.93 ± 0.28	1.72 ± 0.78	1.46 ± 0.76	<0.0001
Median (25th-75th percentile)	0.9 (0.655-1.1)	1.6 (1.2-2.35)	1.2 (0.9-1.8)	
Range	0.6-1.5	0.68-3.6	0.6-3.6	
Height (cm)				
Mean ± SD	167.62 ± 6.37	166.11 ± 6.7	166.62 ± 6.61	0.24^†^
Median (25th-75th percentile)	169.5 (163.5-173)	169 (160-171.5)	169 (160-172)	
Range	155-176	154-177	154-177	
Weight (kg)				
Mean ± SD	60.98 ± 5.42	65.19 ± 6.42	63.78 ± 6.4	0.0004^*^
Median (25th-75th percentile)	61 (58.25-64)	65 (60-69.5)	63 (59-68)	
Range	50-71	54-80	50-80	
HbA1c (%)				
Mean ± SD	8.65 ± 1.46	11.47 ± 2.57	10.52 ± 2.62	<0.0001
Median (25th-75th percentile)	8.45 (7.35-9.55)	11.7 (9.05-14)	9.7 (8.4-13)	
Range	6.8-11.9	7.2-15.2	6.8-15.2	
VAI				
Mean ± SD	1.18 ± 0.59	2.95 ± 1.83	2.35 ± 1.74	<0.0001
Median (25th-75th percentile)	1.08 (0.636-1.512)	2.51 (1.429-4.376)	1.74 (0.994-3.151)	
Range	0.52-2.48	0.68-6.89	0.52-6.89	

**Figure 2:**
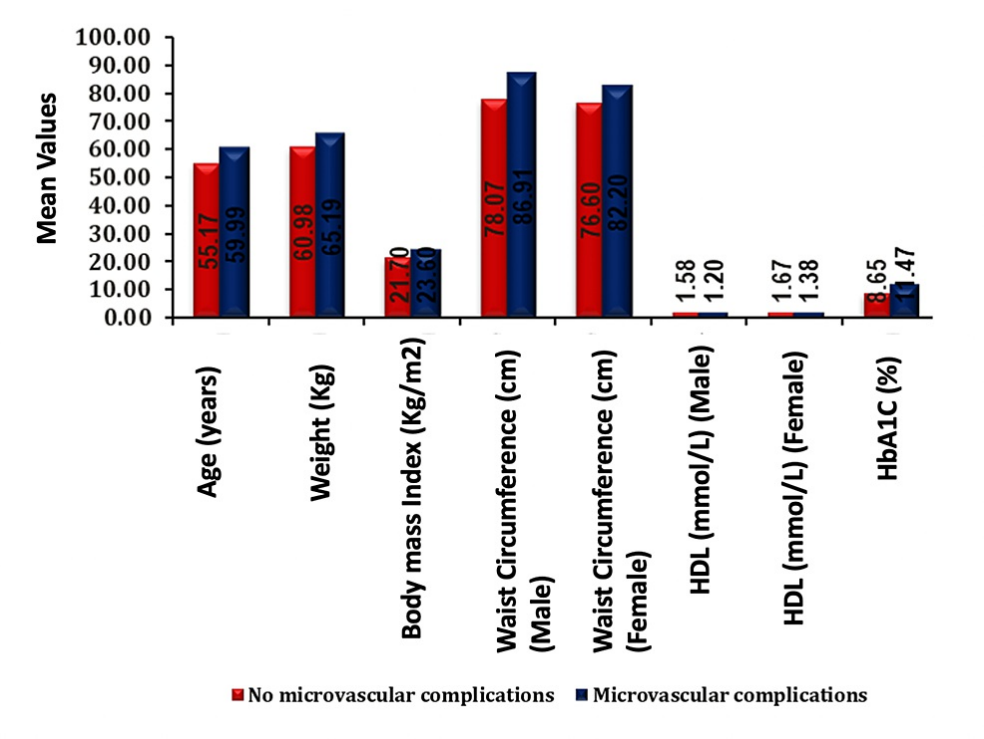
Association of parameters with microvascular complications (mean values). HDL, high-density lipoprotein; HbA1c, glycated hemoglobin

The bar graph showing association of microvascular complications with age, weight, BMI, WC, HDL, and HbA1c in males and females (Figure 2).

**Figure 3:**
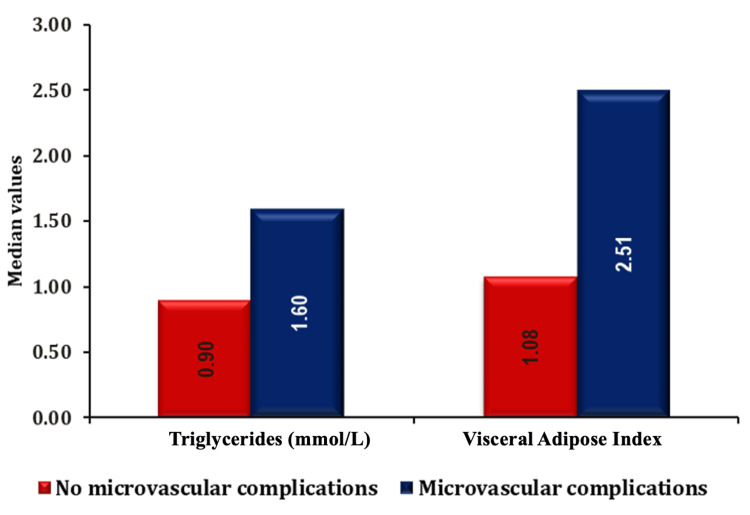
Association of triglycerides and VAI parameters with microvascular complications. VAI, visceral adiposity index

The association of TGs and VAI with microvascular complications is shown in Figure 3.

Table 9 shows various variables of VAI, WC (cm), and BMI (kg/m²) for the prediction of microvascular complications.

**Table 9:** Characteristic variables of VAI, WC (cm), and BMI (kg/m²) for predicting microvascular complications. BMI, body mass index; CI, confidence interval; NPV, negative predictive value; PPV, positive predictive value; ROC, receiver operating characteristic; VAI, visceral adiposity index; WC, waist circumference

Variables	VAI	WC (cm)	BMI (kg/m²)
Area under the ROC curve (AUC)	0.826	0.813	0.806
Standard error	0.036	0.0386	0.0428
95% CI	0.748-0.888	0.733-0.877	0.725-0.871
P-value	<0.0001	<0.0001	<0.0001
Sensitivity (95% CI)	67.47% (56.3-77.4%)	87.95% (79.0-94.1%)	68.67% (57.6-78.4%)
Specificity (95% CI)	83.33% (68.6-93.0%)	54.76% (38.7-70.2%)	78.57% (63.2-89.7%)
PPV (95% CI)	88.9% (78.4-95.4%)	79.3% (69.6-87.1%)	86.4% (75.7-93.6%)
NPV (95% CI)	56.5% (43.3-69.0%)	69.7% (51.3-84.4%)	55.9% (42.4-68.8%)
Diagnostic accuracy	72.80%	76.80%	72%

**Figure 4:**
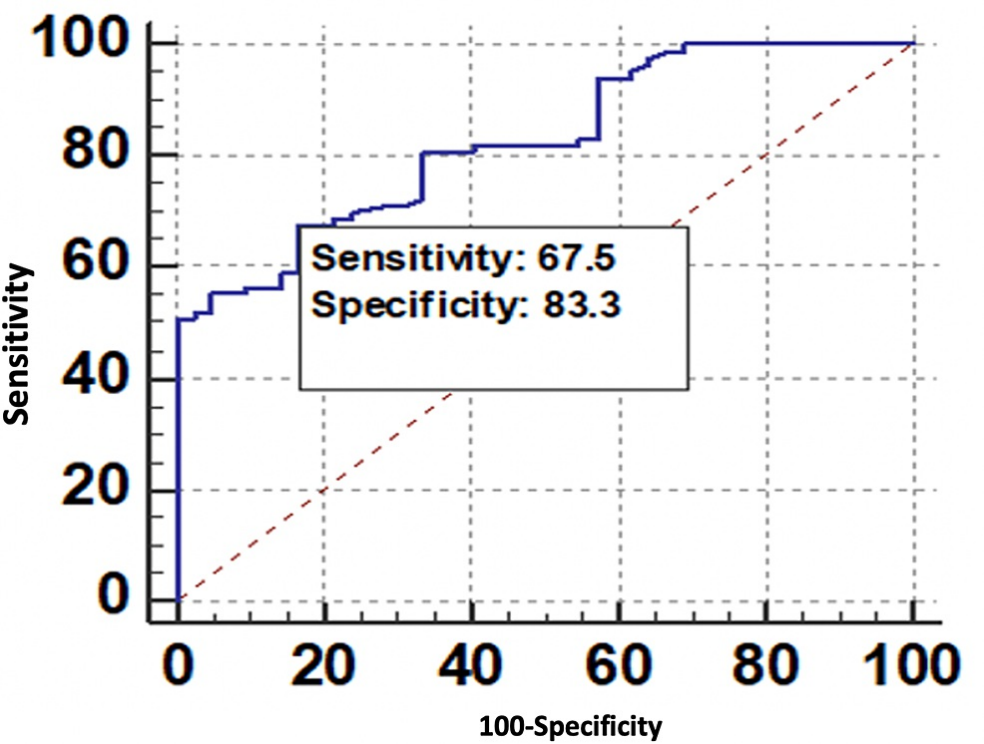
ROC curve of VAI for predicting microvascular complications. ROC, receiver operating characteristic; VAI, visceral adiposity index

The ROC curve of VAI for predicting microvascular complications is shown in Figure 4.

**Figure 5:**
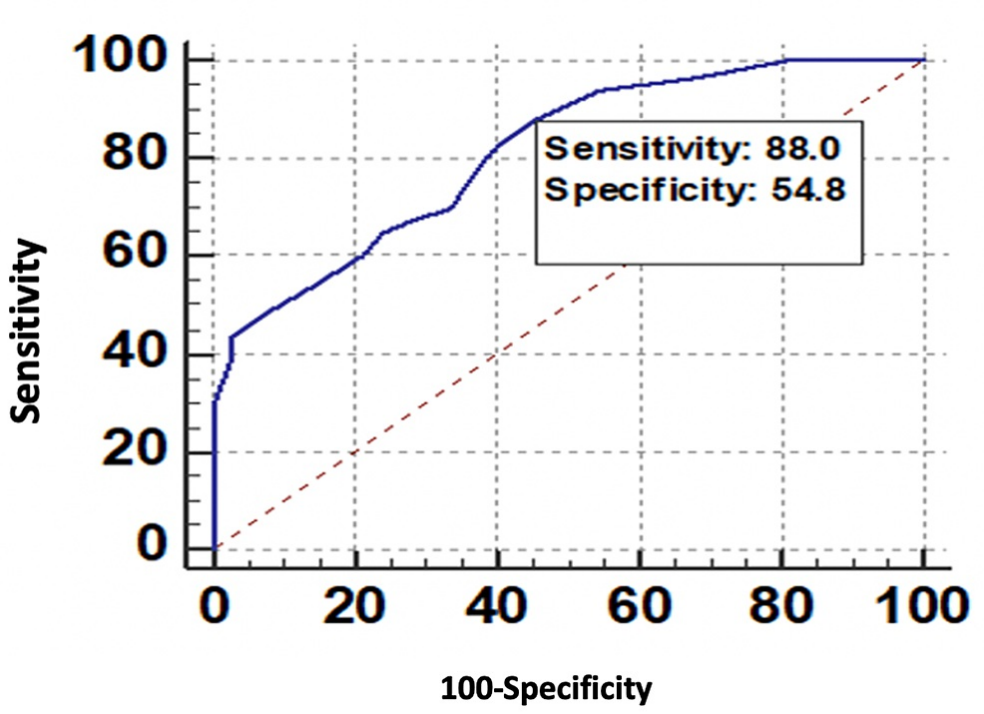
ROC curve of WC (cm) for predicting microvascular complications. ROC, receiver operating characteristic; WC, waist circumference

The ROC curve of WC for predicting microvascular complications is shown in Figure 5.

**Figure 6:**
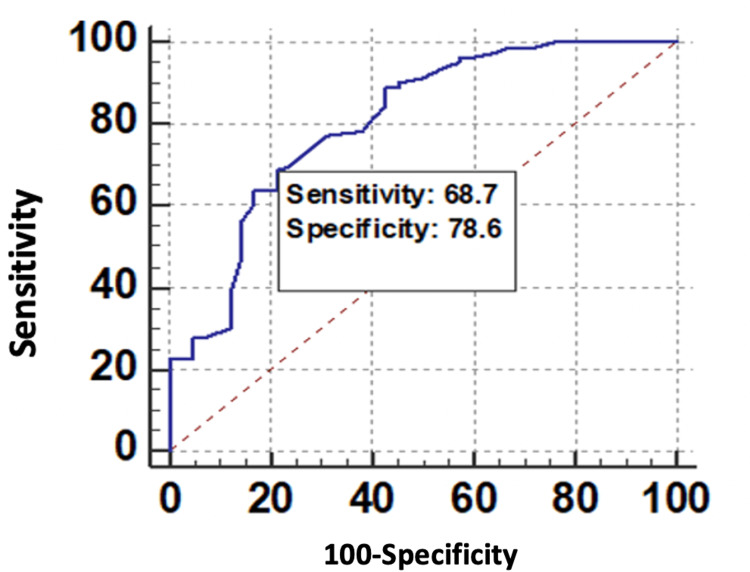
ROC curve of BMI (kg/m²) for predicting microvascular complications. BMI, body mass index; ROC, receiver operating characteristic

The ROC curve of BMI (kg/m²) for predicting microvascular complications is shown in Figure 6.

Univariate logistic regression to find out significant risk factors of microvascular complications is given in Table 10. 

**Table 10:** Univariate logistic regression to find out significant risk factors of microvascular complications. BMI, body mass index; VAI, visceral adiposity index; WC, waist circumference; HDL, high-density lipoprotein; HbA1c, glycated hemoglobin

Variables	Beta coefficient	Standard error	P-value	Odds ratio	Odds ratio lower bound (95%)	Odds ratio upper bound (95%)
Age (years)	0.033	0.016	0.042	1.034	1.001	1.068
Waist circumference (cm)	0.203	0.040	<0.0001	1.225	1.132	1.325
HDL (mg/dL)	–4.785	0.960	<0.0001	0.008	0.001	0.055
Triglyceride (mg/dL)	2.759	0.610	<0.0001	15.790	4.775	52.215
HbA1c (%)	0.584	0.123	<0.0001	1.793	1.408	2.284
VAI	1.291	0.300	<0.0001	3.638	2.020	6.552
BMI (kg/m²)						
18.5-22.99 (Normal)				1.000		
23-24.99 (Overweight)	1.535	0.448	0.001	4.640	1.928	11.167
>25 (Obese)	3.773	1.495	0.012	43.491	2.323	814.151

## Discussion

The study was conducted in AVBRH, a tertiary care teaching hospital situated in the rural area of Wardha District, with 250 Individuals.

In this study, it was observed that the VAI in individuals with T2DM was significantly raised compared with that in nondiabetic control subjects. Moreover, VAI was more raised in diabetic subjects with microvascular complications than those without microvascular complications.

A total of 250 individuals were included in this study of which 125 individuals who had T2DM were enrolled as cases and 125 nondiabetic, age- and gender-matched individuals were enrolled as controls.

Each of the two groups had 70 male subjects (56%) and 55 female subjects (44%). The mean age was 58.37 ± 12.08 and 57.61 ± 14.51 years in cases and controls, respectively.

Both these groups were age- and gender-matched to minimize any influence of age and gender on the results while comparing the two groups.

All the cases enrolled were subjected to inclusion and exclusion criteria. Individuals with T2DM who were either known cases on treatment or newly diagnosed individuals according to the WHO criteria and aged 18 years or more and gave consent to participate in the study were enrolled as cases. Of both the groups, individuals with any nondiabetic nephropathy, hypertension, ocular disorders, and neuropathy in chronic kidney disease; alcoholics; and drug addicts were excluded.

Patients of type 1 DM were not included due to its lower incidence in patients in this hospital as seen from the hospital records. Besides, these individuals have an earlier age of onset and are usually undetected for a longer duration than individuals with T2DM. Type 1 DM has different etiopathogenesis, and a different study based on these subjects would provide more accurate observations. Hence, only patients with T2DM were included in this study.

Microvascular problems, retinal lesions, microalbuminuria and proteinuria, and neuropathies have all been identified as risk factors for cardiovascular (CV) and cerebrovascular morbidity and mortality in diabetics [13]. As a result, if microvascular issues are recognized early, we will be alerted to the increased risk of CV and cerebrovascular complications. As a result, microvascular problems were chosen as the focus of this study.

The VAI is a sex-specific scoring system based on WC, BMI, TGs, and HDL and has the potential of providing knowledge regarding visceral adipose tissue function and insulin sensitivity. It has recently been recommended as a surrogate of visceral adiposity. The VAI, lately introduced by the AlkaMeSy Study Group, can be used as a tool for both visceral fat dysfunction and an individual’s subsequent cardiometabolic risk [12].

It is reported in several studies that various CV risk factors are accountable for prehypertension, particularly obesity. An association is reported between VAI and CV events; this association is better than traditional obesity indices. VAI is found to be an independent risk factor for prediabetes as well as diabetes. Even it is reported that VAI can provide a substitute for visceral CT scanning as a marker for visceral adiposity. The accumulation of visceral adiposity might be antecedent to the onset of metabolic disorders, such as hypertension, T2DM, and insulin resistance, which suggest that VAI can be an early indicator for CV events as well as metabolic syndrome. The potential mechanism behind this is the proliferation of smooth muscle as well as an inflammatory response associated with adiposity stimulation and arteriosclerosis [15].

Association of VAI with DM

The findings of our study were in line with the notion that VAI was significantly higher in diabetic patients as compared with controls (2.35 ± 1.74 versus 1.56 ± 0.8, *P *< 0.005). Thus, there was a positive correlation between VAI and DM.

For females, the median (25th-75th percentile) of VAI in the fourth quartile (>2.609; 3.86 [3.121-5.155]) was the highest followed by the third quartile (1.497-2.609; 1.85 [1.645-2.351]) and the second quartile (0.9625 to <1.496; 1.38 [1.131-1.404]) and the median (25th-75th percentile) of VAI in the first quartile (<0.9625; 0.77 [0.666-0.844]) was the lowest (*P *< 0.0001).

For males, the median (25th-75th percentile) of the VAI in the fourth quartile (>3.5101; 5.6 [5.056-6.166]) was the highest followed by the third quartile (1.9477 to 3.5101; 2.51 [2.354-2.966]) and the second quartile (1.061 to 1.9476; 1.51 [1.251-1.784]) and the median (25th-75th percentile) of VAI in the first quartile (<1.061; 0.69 [0.61-0.769]) was the lowest (*P* < 0.0001).

The results of our study were concordant with some similar studies, as shown in Table 11.

**Table 11:** Comparison of studies showing similar results. AUC, area under curve; BMI, body mass index; CI, confidence interval; DM, diabetes mellitus; ROC, receiver operating characteristic; T2DM, type 2 diabetes mellitus; VAI, visceral adiposity index; WC, waist circumference

No.	Authors	Title	Years	Location	Design	Result
1	Tsou et al. [16]	Visceral adiposity index outperforms conventional anthropometric assessments as predictor of diabetes mellitus in elderly Chinese: a population-based study	2021	Mackay Memorial Hospital, a tertiary teaching center	Population-based study	Chinese VAI shows that the highest discriminatory ability for DM with the area under ROC curves (AUC) of 0.65, 0.68, and 0.66 for men, women, and all participants, respectively, compared with the body shape index, both Chinese VAI and VAI were strongly associated with baseline DM (adjusted odds ratio: 4.85, 95% CI: 4.05-5.82 and 4.22, 95% CI: 3.53-5.05 for fourth versus first quartile groups by CVAI and VAI, *P* < 0.001, which was more pronounced in older adult women (*P*_interaction_ < 0.05).
2	Liu et al. [17]	Visceral adiposity index is associated with pre-diabetes and type 2 diabetes mellitus in Chinese adults aged 20-50	2016	China (2754)	Cross-sectional study	The ROC analysis and AUC results revealed that VAI had the highest AUC, followed by others. In males, the occurrence of diabetes in the highest tertile of the VAI was 2.176 (95% CI 1.404-3.374), *P* = 0.001, while in females, the presence of diabetes was 7.630 (95% CI 2.502-23.268), *P* = 0.001.
3.	Du et al. [18]	Visceral adiposity index, hypertriglyceridemic waist and risk of diabetes: The China Health and Nutrition Survey 2009	2014	China (7639)	Cross-sectional study	In comparison to the first quartile, the age-adjusted odds ratios (95% CI) for diabetes were 1.1 (0.7-1.6), 1.9 (1.3-2.8), and 3.9 (2.8-5.6) for the second, third, and fourth VAI quartiles, respectively. The equivalent statistics for women were 0.9 (0.6-1.5), 1.9 (1.3-2.8), and 3.6 (2.5-5.3).
4	Hameed and AbdulQahar [19]	Visceral adiposity index in female with type 2 diabetic mellitus and its association with the glycemic control	2019	Iraq (300)	Cross-sectional study	AUC for VAI versus WC versus BMI (0.670 versus 0.540 versus 0.491)
5	Chen et al. [20]	The application of visceral adiposity index in identifying type 2 diabetes risks based on a prospective cohort in China	2014	China (4631)	Prospective cohort	The VAI with the highest risk of diabetes was 2.55 times higher (95% CI 1.58-4.11). VAI had the highest AUC, followed by the WC, waist-hip ratio, and BMI. AUC VAI versus WC; 0.62 versus 0.55, P < 0.001.
6.	Wang et al. [21]	Predictive value of visceral adiposity index for type 2 diabetes mellitus: a 15-year prospective cohort study	2015	China (687)	Prospective cohort (15 years)	The HRs were 1.538 (95% CI 1.225-1.930), 1.639 (95% CI 1.289-2.084), and 1.858 (95% CI 1.458-2.369) for each SD rise in natural logarithm-transformed VAI, BMI, and WC, respectively.
7.	Zhang et al. [22]	4-year trajectory of visceral adiposity index in the development of type 2 diabetes: a prospective cohort study	2016	China (4078)	Prospective cohort (4 years)	In the highest tertile of VAI scores, the multivariable-adjusted hazards ratios for developing T2DM were 2.854 (95% CI 1.815-4.487) in males and 3.551 (95% CI 1.586-7.955) in females. AUC VAI versus WC (male: 0.641 versus 0.624; female 0.7171 versus 0.724).

Association of other anthropometric parameters

BMI

Our study suggested that BMI is significantly associated with DM. We found that cases had significantly higher BMI (22.96 ± 1.73 in cases versus 22.51 ± 1.67 in control; *P* < 0.036).

In females, the mean BMI gradually increased through the quartile (Q1, 21.96 ± 1.61; Q2, 21.99 ± 1.01; Q3, 24.14 ± 1.19; and Q4, 24.44 ± 1.63), with *P* < 0.0001.

In males, the mean BMI gradually increased through the quartile (Q1, 20.84 ± 0.84; Q2, 22.44 ± 1.1; Q3, 23.39 ± 1.01; and Q4, 24.56 ± 0.9), with *P* < 0.0001.

Although BMI showed a significant association with diabetes, it suffers from limitations in being used as a marker for DM. It cannot distinguish fat mass from the fat-free mass. Also, it may incorrectly estimate the risk of obesity-related diseases among individuals with heavy muscle mass. Thus, it can be used as an adjunct for the screening of diabetes [23].

BMI is the most widely used measure for determining obesity. It has several advantages as a surrogate for body fat, including simplicity and reliability, and epidemiologic studies have found a link between excessive BMI levels and increased mortality. However, BMI's failure to reflect true body fatness and identify the risk of obesity-related disorders in people with low muscle and high body fat, particularly in people with increased body fat and a normal BMI, is a serious drawback [23].

The findings of our study were in line with the study conducted by Tsou et al. that suggested that BMI was significantly associated with DM outcomes when the various anthropometric parameters were compared with *P* < 0.001 for BMI. However, on compiled analysis, VAI had the maximum AUC (0.66) compared with BMI [16].

Our findings back up Hameed and AbdulQahar's findings, which found that VAI had a high predictive ability to detect the status of glycemic control when compared with other anthropometric measures (WC and BMI) or combined metabolic and anthropometric measurements in 300 T2DM women aged 25-60 years. The study showed that although both are significant, AUC for VAI = 0.670 and AUC for BMI = 0.491, emphasizing VAI is a better predictor for diabetes [19].

WC

According to phase 1 of the study by Pradeepa et al. [24], the incidence of abdominal obesity for the states Tamil Nadu, Maharashtra, and Jharkhand and for the union territory of Chandigarh was found to be 26.6%, 18.7%, 16.9%, and 36.1%, respectively. The prevalence of abdominal obesity assessed by WC was 44.5% in women versus 28% in men, with *P *< 0.001. Multiple regression analysis in this study showed a positive correlation between abdominal obesity and diabetes in these four regions.

Undavalli et al. [25] studied the prevalence of abdominal obesity in India. This study concluded that the incidence of abdominal obesity was 71.2%.

It is known that visceral and central abdominal fat and WC show a strong association with T2DM in India [25]. Abdominal obesity is considered to be a better indicator of underlying complications as compared to BMI [26].

In our study, we found that cases had significantly higher WC 83.5 ± 6.77 for males and 80.67 ± 5.99 for females compared to 80.04 ± 5.43 in male controls and 77.13 ± 5.85 in female controls, respectively, with *P* < 0.001 in males and *P* < 0.002 in females, respectively.

In females, the mean WC gradually increased through the quartile (Q1, 75.07 ± 3.77; Q2, 76.92 ± 3.12; Q3, 84.36 ± 3.89; and Q4, 86.07 ± 4.12), with *P* < 0.0001.

In males, the mean WC gradually increased through the quartile (Q1, 75.44 ± 3.68; Q2, 80.76 ± 3.36; Q3, 87 ± 2.72; and Q4, 90.83 ± 3.11), with *P* < 0.0001.

Hence, with a progressive increase in the quartiles, there was a significant increase in mean BMI, with *P *<= 0.0001.

Our findings were concurrent with the study conducted by Tsou et al. that showed that WC was significantly associated with patients with DM. With WC (*r *= 0.97, *P *< 0.001) in men and women (*r *= 0.83, *P *< 0.001), WC showed an association with DM. However, the AUC suggested that VAI is better than WC [16].

Another study by Liu et al. reported that the *P*-value of WC was <0.002 in males and 0.128 in females compared to *P *< 0.001 of VAI for both males and females. ROC analysis showed that although WC is significant in males, the AUC for VAI is greater than WC [17].

Correlation of diabetes with lipid profile

HDL

In the study, it was observed that HDL was significantly raised (*P* = 0.017) for males in cases compared with control, with mean = 1.35 ± 0.32 in cases as compared to mean = 1.48 ± 0.32 in control. Similarly, HDL was significantly raised (*P *= 0.0004) for females in cases compared to control, with mean = 1.46 ± 0.24 in cases as compared to mean = 1.62 ± 0.22 in control.

The association of HDL with the VAI quartile for females in our cases suggested that the median (25th-75th percentile) of HDL (mg/dL) in the first quartile (<0.9625; 1.66 [1.515-1.852]) was highest followed by the second quartile (0.9625 to 1.496; 1.6 [1.5-1.6]) and the third quartile (1.497 to 2.609; 1.4 [1.3-1.4]) and median (25th-75th percentile) of HDL (mg/dL) in the fourth quartile (>2.609; 1.25 [1.025-1.3]) was lowest (*P *<= 0.0001).

Similarly, the association of HDL with the VAI quartile for males in our cases suggested that the median (25th-75th percentile) of HDL (mg/dL) in the first quartile (<1.061; 1.75 [1.7-1.9]) was highest followed by the second quartile (1.061 to 1.9476; 1.4 [1.3-1.5]) and the third quartile (1.9477 to 3.5101; 1.2 [1.1-1.2]) and median (25th-75th percentile) of HDL (mg/dL) in the fourth quartile (>3.5101; 1 [0.9-1.1]) was lowest (*P *< 0.0001).

TGs

Also, the association of TGs with the VAI quartile for females in our cases suggested that the median (25th-75th percentile) of TGs (mg/dL) in the first quartile (<0.9625; 0.69 [0.68-0.732]) was lowest followed by the second quartile (0.9625-1.496; 1.1 [1-1.2]) and the third quartile (1.497-2.609; 1.35 [1.225-1.575]) and the median (25th-75th percentile) of the fourth quartile (>2.609; 2.45 [2.125-2.7]) was highest (*P* <= 0.0001).

Similarly, the association of TGs with the VAI quartile for males in our cases suggested that the median (25th-75th percentile) of TGs (mg/dL) in the first quartile (<1.061; 0.68 [0.61-0.7]) was lowest followed by the second quartile (1.061-1.9476; 1.1 [1-1.2]) and the third quartile (1.9477-3.5101; 1.5 [1.4-1.7]) and the median (25th-75th percentile) of the fourth quartile (>3.5101; 2.75 [2.325-3]) was highest (*P* < 0.0001).

Thus, the study was corroborative that HDL and TGs were significantly associated with VAI and, thus, the complications of T2DM.

The interpretation of this study is concurrent with the study by Chamba et al., to study the prevalence of abnormal lipid profile levels among patients attending the diabetic clinic for which 119 diabetic patients were included [27]. The study concluded that the prevalence of dyslipidemia was 83% [27].

ZODIAC-13, a 10-year follow-up study conducted by Hateren et al. from 1998 to 2008, suggested the association of lipid profile with the mortality risk of DM patients. The results of the study suggested that HDL (*P *= 0.016) and TGs (*P *<= 0.001) were significantly associated with microvascular complications in DM patients marked by microalbuminuria and altered serum creatinine values [28].

Similar findings were reported by Liu et al. who reported that there were significant differences in TG and HDL cholesterol (HDLC) in cases. In males, the upper VAI tertile participants exhibited lower HDLC (mmol/L; 1.31 versus 1.15 versus 1.04, *P *< 0.001), higher triacylglycerols (mmol/L; 0.94 versus 1.68 versus 2.97, *P *< 0.001), compared with the lower VAI subjects. In females also, the upper VAI tertile participants exhibited lower HDLC (mmol/L; 1.62 versus 1.40 versus 1.18, *P *< 0.001), higher triacylglycerols (mmol/L; 0.61 versus 0.99 versus 1.81, *P *< 0.001) compared with the lower VAI subjects [17].

Consistent with this, Tsou et al. observed that in comparison with the low-quartile group, the upper VAI quartile participants had significantly higher TG (mmol/L; 0.79 versus 0.99 versus 1.25 versus 1.41, *P *< 0.001) and significantly lower HDLC (mmol/L; 1.56 versus 1.30 versus 1.20 versus 1.08, *P *< 0.001) in males compared to females. In females also, the upper VAI quartile participants showed lower HDLC (mmol/L; 1.83 versus 1.56 versus 1.42 versus 1.29, *P *< 0.001) and higher triacylglycerols (mmol/L; 0.79 versus 1.10 versus 1.32 versus 1.63, *P *< 0.001) [16].

Du et al. reported that as the VAI quartile increased, the HDL was inversely related and TGs were directly associated with the risk of DM. HDLC (mmol/L; 1.7 versus 1.4 versus 1. 2 versus 1, *P* < 0.001) for males and HDLC (mmol/L; 1.7 versus 1.5 versus 1.4 versus 1.2, *P *< 0.001) for females and TGs (mmol/L; 0.7 versus 1.1 versus 1.5 versus 2.7, *P *< 0.001) for males and (mmol/L; 0.7 versus 1.0 versus 1.4 versus 2.4, *P *< 0.001) for females [18].

Even, Wu et al. reported that individuals of upper quartile groups had significantly higher TGs (mmol/L; 0.96 ± 0.45 versus 1.29 ± 0.57 versus 1.64 ± 0.79 versus 1.91 ± 0.85, *P *< 0.001), low-density lipoprotein cholesterol (LDLC, mmol/L; 2.32 versus 2.54 versus 2.58 versus 2.57), and significantly lower HDLC (mmol/L; 1.26 ± 0.26 versus 1.17 ± 0.23 versus 1.10 ± 0.22 versus 1.04 ± 0.20, *P *< 0.001) [29].

Excessive visceral fat deposition, particularly abdominal fat, causes physiological changes that result in a disrupted lipid profile. Obesity, particularly central obesity, is a critical factor in the development and progression of diabetes as this deranged lipid profile is associated with DM and attributed to increased free fatty acid flux secondary to insulin resistance [30].

Overall, the relationship between visceral adipose tissue and the risk of DM occurrence may vary in different countries and ethnic groups. However, VAI can act as a simpler and more economical index to evaluate visceral adipose and the risk of diabetes.

The Quebec Cardiovascular Study group was the first to identify the *hypertriglyceridemic waist phenotype* as a marker of excess visceral adiposity and the atherogenic metabolic triad (i.e., hyperinsulinemia, hyperapolipoprotein B, and small, dense LDL particles) in men, demonstrating that the hypertriglyceridemic waist phenotype was a better marker and better predictor of CV risk, whereas the CHICAGO cohort discovered that in those with T2DM, the hypertriglyceridemic waist phenotype could be a simple indication of extra visceral fat [31]. Several studies found consistent favorable relationships between the hypertriglycemic waist phenotype and the risk of coronary heart disease.

Our findings reveal a strong link between the hypertriglyceridemic waist phenotype and the risk of diabetes. An individual's CV disease risk is linked to their body size and metabolic profile, according to studies [32].

Our discovery that the hypertriglyceridemic waist phenotype, which indicates the presence of abdominal obesity and metabolic abnormalities at the same time, confers a higher diabetes risk than merely increased WC or solely increased TG, adds to this theory.

Association of VAI, WC (cm), and BMI (kg/m²) for predicting microvascular complications

In our study, on comparing the study population with and without microvascular complications, BMI, WC, VAI, HDL, TGs, and HbA1c were found to be significantly associated with the microvascular complications of DM.

In this study, the ROC curve analysis indicated that of the various obesity indices tested, the AUC values for the VAI, WC, and BMI were 0.826, 0.813, and 0.806, respectively. Although all the anthropometric parameters have a significant association with microvascular complications in diabetic patients (*P *> 0.001), VAI with the maximum AUC and highest specificity of 83.33% (68.6%-93.0%) was an ideal tool compared to WC and BMI (VAI > WC > BMI).

The results of this study for predicting microvascular complications were concordant with some similar studies, as shown in Table 12.

**Table 12:** The various studies that compared the different microvascular complications with various anthropometric parameters. BMI, body mass index; CKD, chronic kidney disease; CI, confidence interval; DN, diabetic neuropathy; DR, diabetic retinopathy; DPN, diabetic peripheral neuropathy; OR, odds ratio; VAI, visceral adiposity index; WC, waist circumference; WHtR, waist-to-height ratio

No.	Author	Title	Year	result
1	Moh et al. [33]	Excess visceral adiposity is associated with diabetic retinopathy in a multiethnic Asian cohort with longstanding type 2 diabetes	2018 (n = 953)	BMI, WC, and VAI were all higher in DR than in non-DR patients with T2D (all *P* = 0.05). Unlike BMI and WC, the link between VAI and DR was maintained after accounting for demographics, metabolic variables, and insulin therapy (OR = 1.060, 95% CI 1.004-1.119, *P* = 0.035).
2.	Zhou et al. [34]	Is central obesity associated with diabetic retinopathy in Chinese individuals? An exploratory study	2019 (n = 511)	According to the meta-analysis, central obesity raised the incidence of DR by 12% (OR 1.12, 95% CI 1.02-1.22). The results of an analysis of data from 18 studies revealed a significant link between continuous BMI and the probability of proliferative DR (OR 0.95, 95% CI 0.93-0.98; I2 = 50%).
3.	Wan et al. [35]	Associations between abdominal obesity indices and diabetic complications: Chinese visceral adiposity index and neck circumference	2020 (n = 4,813)	The area under the ROC curve of BMI, WC, and VAI for DKD was 0.568, 0.588, and 0.561, respectively (all *P* < 0.05).
4.	Bamba et al. [36]	The visceral adiposity index is a predictor of incident chronic kidney disease: a population-based longitudinal study.	n = 1,078	The area under the curve of VAI for incidence of CKD was superior to that of VAI in men (0.595 versus 0.552, P < 0.001) and equal to in women (0.597 versus 0.591, *P* = 0.708).
5.	Hukportie et al. [37]	Anthropometric measures and incident diabetic nephropathy in participants with type 2 diabetes mellitus	2021 (n = 8,887)	In the multivariate analysis, null relationships were found between all anthropometric parameters with incident DN in men, while the third quartile of WHtR had marginally significant results (*P* = 0.052). However, both central and general obesity measurements were linked to an elevated risk of incident DN in women. The completely adjusted HR and 95% CI for those in the WC = 88 cm category were 1.35 compared to those in the WC = 88 cm group (95% CI 1.15-1.57). The fully adjusted HRs and 95% CIs for the second to fourth quartiles of WHtR were 1.09 (95% CI 0.96-1.25), 1.12 (95% CI 0.98-1.28), and 1.14 (95% CI 1.00-1.30), respectively, when compared to the lowest quartile; also, when compared to the normal BMI category, the fully adjusted HRs and 95% CIs for classes I-III obese were 1.
6.	Oh et al. [38]	Association between body fat and diabetic peripheral neuropathy in middle-aged adults with type 2 diabetes mellitus	2019 (n = 65)	DPN was linked to WC (OR 1.151; 95% CI 1.055-1.256; *P* = 0.002), visceral fat area (OR 1.026; 95% CI 1.005-1.048; *P* = 0.015), and insulin resistance (OR 1.673; 95% CI 1.091-2.565; *P* = 0.018). Subjects with DPN had a greater BMI and WC than those who did not.
7.	Zhou et al. [39]	Associations between general and abdominal obesity and incident diabetic neuropathy in participants with type 2 diabetes mellitus	2021 (n = 7,442)	In both men and women, larger WCs were linked to a higher risk of DN. In men, the hazard ratio (HR) for the top quartile was 1.30 (95% CI 1.13-1.49) when compared to the lowest quintile (*P*_trend_ 0.001). The HR between the highest and lowest quintiles for women was 1.25 (95% CI 1.04-1.51; *P*_trend_ 0.001). In both men and women, there was a linear association between WC and DN. The findings for the link between BMI and incidence DN were comparable to those for WC.

On performing univariate regression analysis, WC, HDL (mg/dL), TGs, HbA1c, BMI, and VAI were significant risk factors of microvascular complications, with *P* < 0.0001, *P* < 0.0001, *P* < 0.0001, *P* < 0.0001, *P* = 0.001, and *P* < 0.0001, respectively. With the increase in WC, the risk of microvascular complications increases with an odds ratio of 1.225 (1.132-1.325). With the increase in HDL (mg/dL), the risk of microvascular complications significantly decreases. With the increase in TGs, the risk of microvascular complications significantly increases with an odds ratio of 15.790 (4.775-52.215). With the increase in HbA1c, the risk of microvascular complications increases with an odds ratio of 1.793 (1.408-2.284). With the increase in BMI, the risk of microvascular complications increases with an odds ratio of 4.640 (1.928-11.167). With the increase in VAI, the risk of microvascular complications increases with an odds ratio of 3.638 (2.020-6.552).

Limitations

In this study, the VAI was established in specific geographical locations in the Indian population; however, its suitability for populations of other countries requires to be further evaluated. Thus, being a single-center hospital-based study, its results cannot be generalized. Follow-up was not done for the patients. Adjustment of other potential confounding factors such as dietary patterns and blood pressure was not done.

## Conclusions

It can be concluded that VAI is significantly increased in diabetics as compared to nondiabetics, showing a significant correlation with deranged lipid profile (parameters like TG and HDL) and anthropometric parameters like BMI and WC. The study findings suggest that VAI can be a useful marker for monitoring a patient’s progression as the patient was studied for the microvascular complications associated with type 2 DM. The study also highlighted that in patients with raised HbA1c showed a higher risk for the development of microvascular complications such as retinopathy, nephropathy, and neuropathy. Hence, VAI along with the WC is an excellent tool for predicting microvascular complications.
